# Lipid Metabolism Profiles in Rheumatic Diseases

**DOI:** 10.3389/fphar.2021.643520

**Published:** 2021-04-09

**Authors:** Weilin Chen, Qi Wang, Bin Zhou, Lihua Zhang, Honglin Zhu

**Affiliations:** ^1^ Department of Rheumatology, Xiangya Hospital, Central South University, Changsha, China; ^2^ National Clinical Research Center for Geriatric Disorders, Xiangya Hospital, Changsha, China; ^3^ Provincial Clinical Research Center for Rheumatic and Immunologic Diseases, Xiangya Hospital, Changsha, China; ^4^ Department of Radiology, Hunan Provincial People's Hospital, The First Affiliated Hospital of Hunan Normal University, Changsha, China; ^5^ Department of Nephrology, The Affiliated Hospital of Qingdao University, Qingdao, China; ^6^ Department of Rheumatology, Hunan Provincial People's Hospital, The First Affiliated Hospital of Hunan Normal University, Changsha, China

**Keywords:** dyslipidemia, fatty acid metabolism, rheumatic diseases, lipid metablism, statin

## Abstract

Rheumatic diseases are a group of chronic autoimmune disorders that involve multiple organs or systems and have high mortality. The mechanisms of these diseases are still ill-defined, and targeted therapeutic strategies are still challenging for physicians. Recent research indicates that cell metabolism plays important roles in the pathogenesis of rheumatic diseases. In this review, we mainly focus on lipid metabolism profiles (dyslipidaemia, fatty acid metabolism) and mechanisms in rheumatic diseases and discuss potential clinical applications based on lipid metabolism profiles.

## Introduction

Rheumatic diseases are a group of chronic heterogeneous autoimmune disorders that involve multiple organs or systems and cause high mortality and disability. The major rheumatic diseases include systemic lupus erythematous (SLE), rheumatoid arthritis (RA), systemic sclerosis (SSc), idiopathic inflammatory myopathy (IIM), and Sjögren's syndrome (pSS). The pathogenesis of rheumatic diseases is complicated and poorly defined.

Recently, immunometabolism has been widely studied in autoimmune and rheumatic diseases, and studies have mainly focused on six major metabolic pathways, including glycolysis, the tricarboxylic acid (TCA) cycle, the pentose phosphate pathway (PPP), amino acid metabolism, fatty acid (FA) oxidation and FA synthesis. Among these pathways, research into lipid metabolism has been ongoing for years (Rhoads et al., 2017; Peradze et al., 2019). For example, dyslipidaemia was associated with CD4^+^ T cell activation and complement-mediated renal damage in lupus-prone mouse models (Woo et al., 2010; Black et al., 2015). Statin suppressed the secretion of pro-inflammatory cytokines by macrophages and T cells in RA patients (Kwak et al., 2000). In SSc and fibrotic disease, lipid metabolism was a key mediator in the activation of fibroblasts and immune cells. In addition, adipose tissue is correlated with oxidative stress and participates in vascular damage (Winsz-Szczotka et al., 2016).

Dyslipidaemia is characterized by lower high-density lipoprotein (HDL) levels and higher low-density lipoprotein (LDL), triglyceride (TG), and total cholesterol (TC) levels, and it is commonly found in rheumatic diseases. It is well known that high levels of LDL and/or abnormal levels of HDL in the plasma are strongly correlated with an increased risk of atherosclerosis (Expert, 2001) and end-organ damage, such as central nervous system and kidney damage (Tselios et al., 2016). Here, we summarize the profiles of lipid metabolism in rheumatic diseases and explore potential clinical applications.

## Lipid Metabolism Disorders in SLE

SLE is a chronic autoimmune disease that involves multiple organs and is characterized by heterogeneous symptoms (Kaul et al., 2016). A high risk of dyslipidaemia is observed in SLE patients. Hypercholesterinaemia was observed in 36% of newly diagnosed SLE patients from the International Collaborating Clinics cohort, with even higher levels after being diagnosed for 3 years (Urowitz et al., 2007). Dyslipidaemia can affect the prognosis of SLE patients through both cardiovascular disease (CVD)-related events and damage to other organs, such as lupus nephritis (Tselios et al., 2016).

### Dyslipidaemia in SLE

#### High-Density Lipoprotein

Pro-inflammatory HDL (piHDL) was found in 44.7% of female SLE patients and 20.1% of female RA patients but in only 4.1% of healthy women (McMahon et al., 2006). Dysfunctional piHDL notably increased the prevalence of subclinical atherosclerosis and carotid plaque, with higher intima-media thickness (IMT), in SLE patients (Wu et al., 2016), especially in female patients (McMahon et al., 2014). In addition, the occurrence of ischaemic heart disease is 50-fold higher in female SLE patients of childbearing age (Manzi et al., 1997; Manzi et al., 1999). Decreased HDL and Apo A-1 and increased oxidized LDL (ox-LDL) auto-antibody levels were also observed in paediatric SLE patients (Soep et al., 2004; Yuan et al., 2016). Paraoxonase-I (PON-1) is a subfraction of HDL (Durrington et al., 2001), which can protect LDL from oxidation (Mackness et al., 1991). PON-1 might inhibit the synthesis of cholesterol in macrophages and promote HDL reverse cholesterol transport (Aviram and Rosenblat, 2004). The levels of plasma PON-1 are decreased in SLE patients (Kiss et al., 2007), and reduced PON-1 activity may be involved in SLE complications.

It has been widely recognized that HDLs are involved in the anti-inflammatory processes (Saemann et al., 2010). The mechanisms of the anti-inflammatory effects are still elusive. HDL can activate the transcriptional repressor activating transcription factor 3 (ATF-3) thus inhibits Toll-like receptor (TLR) pathways and TLR-induced cytokines (De Nardo et al., 2014). HDL can also inhibit NF-κB mediated vascular inflammation (Park et al., 2003). Compared to control HDL, SLE HDL can activates NF-κB, increase the production of inflammatory cytokine production, decrease ATF3 synthesis and activity in a LOX1R- and ROCK1/2-dependent manner. HDL-targeted therapies can serve as potential therapeutic intervention for SLE patients with CVD (Smith et al., 2017; Kim et al., 2020).

#### Low-Density Lipoprotein

LDL becomes oxidized in the vascular wall and induces monocyte chemotaxis in SLE patients (Hansson, 2005; Narshi et al., 2011). Normal HDL can protect LDL from oxidation *in vivo* (Navab et al., 2004), thus decreasing the risk of CVD in SLE patients (Gaal et al., 2016). Increased LDL and/or ox-LDL levels are positively correlated with plaque inflammation in SLE patients, especially in female patients. Adaptive immune responses might diminish inflammation and accelerate vascular repair (Wigren et al., 2015). Nevertheless, long-term exposure to high levels of LDL will lead to loss of tolerance to ox-LDL antigens (Nilsson and Hansson, 2008). Thus, the intensity of the immune response to ox-LDL may determine the progression of CVD in SLE.

#### Apo A-1 and Anti-Apo Antibody

Apo A-1 is the major lipid-binding protein in HDL. Elevated plasma apo A-1 can significantly repress the activation of cells and the secretion of interferon-γ (IFN-γ) in apo A-1 genetically modified lupus-prone mouse models (Black et al., 2015). Fewer CD4^+^ T cells infiltrated the kidney, and glomerulonephritis was also improved in this model. The administration of apo A-1 analogues can relieve lupus-like manifestations in lupus-prone mouse models (Woo et al., 2010).

Anti-apo A-1 antibodies are commonly found in SLE patients, even very early in the disease course (Croca et al., 2015). The titres of anti-apo A-1 antibody are positively correlated with SLE-related auto-antibodies and the SLE disease activity index (SLEDAI) (Radwan et al., 2014; Croca et al., 2015). Anti-oxLDL and anti-apo B antibodies were observed in primary antiphospholipid syndrome (APS) (Zhao et al., 2001) and SLE patients (Svenungsson et al., 2001). Their levels are much higher in SLE patients with high disease activity (O'Neill et al., 2010). In addition, these antibodies cross-react with anti-cardiolipin (Delgado Alves et al., 2003), indicating a potential interaction between the immune response and lipid metabolism in SLE. Antibodies against lipid components might be novel biomarkers that indicate SLE disease activity (Hahn, 2010).

#### Dyslipidaemia in Lupus Nephritis (LN)

Decreased HDL and Apo B levels as well as increased LDL, TG and TC levels were observed in SLE patients with LN (Liu et al., 2014), even in the quiescent phase (Chong et al., 2011). Disease activity aggravates the abnormal lipid profile course in patients with SLE (Borba and Bonfa, 1997). Dyslipidaemia can enhance the CXCR3^+^ follicular T helper cell (T_FH_ cell) response and promote immunoglobulin IgG2c production in a manner dependent on Toll-like receptor 4 (TLR4) and the cytokine IL-27 in the SLE mouse model (Ryu and Chung, 2018; Ryu et al., 2018). Hyperlipidaemia could amplify complement activation and enhance renal inflammation, thus promoting nephritis, in lupus-prone mouse models (Lewis et al., 2012).

### FA Metabolism in SLE

N-3 polyunsaturated fatty acids (PUFAs) and n-6 PUFAs are reduced, while their downstream products (5-HETE and leukotriene B4) are markedly elevated in serum from SLE patients (Wu et al., 2012). Free fatty acids (FFAs) are common regulators of inflammation, immunity and lipid metabolism (de Jong et al., 2014). Elevated serum FFAs are observed in SLE patients with intestinal dysbiosis, indicating a potential link to the gut microbiota (Rodriguez-Carrio et al., 2017). Evidence shows that both Prostaglandin D2 (PGD2) and Resolvin D1 (RvD1) can restore homeostasis in inflammatory tissues. Lower levels of RvD1 were found in SLE patients (Navarini et al., 2018). PGD2 could aggravate SLE disease by promoting basophil accumulation in the lymph nodes through interactions with the CXCL12-CXCR4 axis, and antagonize PGD2 receptors (PTGDR) can reduce lupus-like disease in induced and spontaneous mouse models (Pellefigues et al., 2018), PGD2/PTGDR axis maybe a ready-to-use therapeutic target in SLE.

### Potential Treatment Based on Lipid Metabolism in SLE

#### Statins

Treatment with statins seems to be beneficial to SLE patients, but the effects are still elusive. Aggressive treatment of dyslipidaemia reduces the risk of lupus nephritis and atherosclerosis (Lewis et al., 2012). In SLE patients, fluvastatin could regulate the lipid metabolism pathway in monocytes and exert anti-oxidative and anti-inflammatory effects (Ruiz-Limon et al., 2015). Atorvastatin can restore T cell signalling and reduce the levels of IL-6 and IL-10 (Jury et al., 2006), but its effects on SLE disease activity were controversial in a double-blind randomized clinical trial (Fatemi et al., 2014). In a 11- year follow-up cohort, statins could reduce the risk of mortality, CVD and end-stage renal disease only in SLE patients with high disease activity (Yu et al., 2015).

#### FA Supplements

Oral fish oil (FO) can upregulate the levels of IL-13, downregulate the levels of IL-12 and restore systemic inflammation in SLE patients (Arriens et al., 2015). N-3 FAs can upregulate adiponectin in SLE patients (Lozovoy et al., 2015). Both FO and N-3 FAs can promote macrophage uptake of apoptotic cells and decrease the levels of CD4^+^ T infiltration in the kidney, with the latter effect leading to relief of renal disease (Itoh et al., 2007; Shirai and Suzuki, 2008). Beyond the recommended doses, DHA and EPA extended the lifespan in a dose-dependent manner, downregulated the levels of anti-dsDNA antibodies and the pro-inflammatory cytokines IL-1β, IL-6, and TNF-α, and attenuated glomerulonephritis in SLE patients (Halade et al., 2013). Peripheral blood mononuclear cells (PBMCs) pre-exposed to EPA or DHA reduced the expression of IL-1β, IL-2 and TNF-α in both SLE patients and healthy controls after stimulation with methylmercury (MeHg). N-3 long-chain PUFAs can reduce the extent of the inflammatory response, and their anti-inflammatory effects are more effective in PBMCs from healthy controls than in PBMCs from SLE patients (Crowe et al., 2018).

## Lipid Metabolism Disorders in RA

RA is a chronic inflammatory autoimmune disease affecting 0.5–1% of the population (Symmons et al., 2002). The disorder of HDL, LDL and TC levels has been reported in the active course of RA (Choy and Sattar, 2009), and a higher prevalence of dyslipidaemia was observed in RA patients (Garcia-Gomez et al., 2009). Moreover, lipid abnormalities are associated with systemic inflammation in RA patients (Dessie et al., 2020). Based on the study of female RA patients and age-matched non-RA female controls, the relationship between lipid metabolism and skeletal muscle mass was found in RA, and it’s independent of disease severity and body fat mass (Matsumoto et al., 2020).

### Dyslipidaemia in RA

HDL anti-oxidant capacity is negatively correlated with RA disease activity. Oxidation rates were 56% higher in RA patients with high inflammation (Gomez Rosso et al., 2014). Pro-oxidant HDL was increased more than five times in RA patients compared to normal controls (McMahon et al., 2006). High levels of HDL were correlated with erythrocyte sedimentation rate (ESR), high sensitivity C-reactive protein (hsCRP) and Disease Activity Score in 28 joints (DAS28) (Charles-Schoeman et al., 2009). A reduction of cholesterol efflux capacity was observed in RA patients with high DAS28 scores (Charles-Schoeman et al., 2012).

The levels of serum ox-LDLs (Kim et al., 2004; Vuilleumier et al., 2010) and anti-apoA-1 IgG (Kim et al., 2004) are also associated with RA disease activity. Increased levels of ox-LDLs were found in synovial fluid (Dai et al., 2000) and synovium (Winyard et al., 1993) and were positively correlated with IMT in RA patients with CVD (Ahmed et al., 2010). In addition, anti-apoA-1 IgG appears to be independent of traditional CVD risk factors and therapeutic effects. Therefore, measurement of lipid profiles and identification of inflammatory status might help us to assess the development of diseases.

Moreover, liver X receptor α (LXRα) mediated key lipogenic enzymes, such as fatty acid translocase (CD36/FAT), lipoprotein lipase (LPL), adipocyte fatty acid-binding protein (aP2/FABP4) and cholesterol 7α and 27α hydroxylase (CYP7A, CYP27 A), can aggravate dyslipidaemia in adjuvant-induced arthritis (Xie et al., 2021). And repress LXRα agonism enables to reverse the dyslipidaemia in RA. This study indicates a potential therapy target to develop new drugs against RA with dyslipidaemia, further mechanisms need to be revealed.

### FA Metabolism in RA

Lower serum FFAs are observed in newly diagnosed RA patients (Young et al., 2013). PGE2 was increased in the synovial fluid of RA patients and altered after treatment. Compared to non-steroidal anti-inflammatory drugs, steroids can elevate PGE2 levels (Hishinuma et al., 1999). High levels of LTB4 were also found in the SF of RA patients (Davidson et al., 1983). The LTB4 secretion capacity of neutrophils was enhanced, suggesting that it may be involved in the pathogenesis of RA (Elmgreen et al., 1987). LTB4 can also mediate the expression of IL-1β and TNFα in RA synovial fibroblasts (RASFs) (Xu et al., 2010) and influence the invasion and migration capacity of RASFs (Chen et al., 2010). Joint administration LTB4 contributes to bone loss by promoting osteoclast activity (Garcia et al., 1996). 15-Lipoxygenase (15-LOX) mRNA was detected in type B synoviocytes of RA patients and participated in the production of 15-HETE, which can be promoted by IL-4 and IL-1β (Liagre et al., 1999). Higher levels of pro-inflammatory cytokine IL-6 and IL-8, monocyte chemotactic 1 and growth-related oncogene α were secreted by FFAs-stimulated osteoblasts from RA patients (Frommer et al., 2019). And no association was found with Wnt signalling pathway and receptor activator of nuclear kappa B ligand (RANKL). Instead, inhibiting TLR-4 can remarkably reduce PA-induced IL-8 secretion, but no effects were found with blocking TLR-2. Thus, the FFA signalling for osteoblasts might be dependent on innate immune system and inflammation.

### Potential Treatments Based on Lipid Metabolism in RA

#### Anti-Rheumatic Drugs

Dyslipidaemia can be reversed by anti-inflammatory and anti-rheumatic drugs in RA patients without using statins (Garcia-Gomez et al., 2009; Steiner and Urowitz, 2009). Glucocorticoid treatment can elevate HDL levels and reduce the risk of CVD (Hahn et al., 2007). However, the activity of cholesteryl ester transfer protein is still low in RA patients treated with glucocorticoid therapy (Ferraz-Amaro et al., 2013). Rituximab and anti-TNF therapies can increase the levels of ox-LDLs temporarily at three months and can also increase the level of Apo A-1 (Ajeganova et al., 2011). RA patients treated with methotrexate (MTX) or MTX combined with anti-TNF (Hjeltnes et al., 2013) or tocilizumab (Schultz et al., 2010) have lower levels of lipoproteins.

#### Statins

Statins can exert anti-inflammatory and anti-oxidative effects in normal controls (McMahon and Brahn, 2008), which can inhibit co-stimulatory factors on the surface of antigen-presenting cells and IFN γ-induced Class II major histocompatibility complex (MHC) antigens on the surface of macrophages (Kwak et al., 2000). Atorvastatin can effectively reduce the anti-inflammatory effects of HDL (Charles-Schoeman et al., 2007). Overdosage of simvastatin can relieve arthritis inflammation in RA mouse models and downregulate the expression of pro-inflammatory cytokines (Leung et al., 2003).

#### FA Supplements

Anti-inflammatory effects of FO were demonstrated in RA (Kremer et al., 1985), especially for reducing the secretion of IL-1 by monocytes, restoring the concentrations of CRP and normalizing the chemotaxis of neutrophils. N-3 long-chain PUFA administration decreased the degree of swelling and the duration of morning stiffness (Berbert et al., 2005). FO and arachidonic acid (AA) supplementation decreased pro-inflammatory factors (LTB4 and prostaglandin metabolites) and improved joint pain in RA patients ((Adam et al., 2003), (Volker et al., 2000)). In addition, daily oral EPA and DHA can help RA patients reduce their NSAID dosage without deterioration of their condition (Galarraga et al., 2008). However, no significant clinical improvement was observed for low-dosage oral EPA and DHA (1.4 ± 0.2 g), and the effects may be dosage dependent (Remans et al., 2004). Meanwhile, activation of fatty acid sensing GPCR (Gpr84) or medium-chain FFAs supplementation are supposed to preventing the progression of osteoarthritis without cartilaginous side effect (Wang et al., 2020).

## Lipid Metabolism Disorders in SSc

SSc is a devasting autoimmune disease that involves vascular damage and progressive fibrosis of internal organs (Denton and Khanna, 2017). Adipose tissue loss and oxidative stress contribute to fibrosis.

### Dyslipidaemia in SSc

Lower levels of HDL-C and higher levels of LDL were found in SSc patients (Tsifetaki et al., 2010), and carotid artery IMTs were also significantly higher. Lipoprotein(a) is synergy with prothrombotic conditions in the pathogenesis of vascular damage in SSc (Lippi et al., 2006). The ox-LDL/β2GPI complex is induced by oxidative stress and participates in autoimmune vascular inflammation in SSc (Lopez et al., 2005).

### Adipose Tissue in SSc

Atrophied intradermal adipose tissue, first observed in 1972, is replaced by fibrosis during skin induration in SSc patients (Fleischmajer et al., 1972). In addition, downregulated adipogenic markers (Wu et al., 2009; Marangoni et al., 2015) peroxisome proliferator activated receptor-γ2 (PPARγ2), fatty acid-binding protein 4 (FABP4) and adiponectin) as well as reduced thickness and total volume of dermal white adipose tissue (dWAT) (Kasza et al., 2016) were detected in skin from scleroderma mouse models. Adipose tissue plays critical roles in the pro-oxidative/anti-oxidative system (Winsz-Szczotka et al., 2016), the latter is also identified as a major cause of vascular damage in SSc (Bruckdorfer et al., 1995; Sambo et al., 2001). Oxidative injury, such as lipid peroxidation, leads to structural and functional disorders of the erythrocyte membrane and contributes to microvascular damage (Solans et al., 2000).

### FAs in SSc

Numerous studies have reported that FAs and their metabolites are involved in fibrosis. Upregulated LTB4 (Kowal-Bielecka et al., 2003; Kowal-Bielecka et al., 2005) and leukotriene E4 (LTE4) (Kowal-Bielecka et al., 2003) were found in bronchoalveolar lavage (BAL) fluid from SSc patients and were revealed to be parameters of inflammation in the lungs. LOX plays a critical role in the process of leukotriene synthesis. 5-LOX-derived leukotrienes were involved in the development of lung fibrosis in bleomycin-induced mouse models (Beller et al., 2004), and the fibrosis index was alleviated in 5-LOX knockout mice (Selman et al., 2004). Unlike the function of the leukotriene subfamily, PGE2 (Wilborn et al., 1995) and Prostaglandin I2 (PGI2) (Soberman and Christmas, 2006) are antifibrogenic, while PGF2α (Oga et al., 2009) is profibrogenic. *In vitro*, PGI2 analogues (iloprost, treprostinil and beraprost) can affect T_H_ cell differentiation programmes and promote T_H_ 17 cell responses in SSc PBMCs (Truchetet et al., 2012). Upregulated PGF2α synthesis promotes the development of fibrosis in a bleomycin-induced mouse model (Kanno et al., 2013).

Murine 12/15-LOX and human 15-LOX are enzymes that regulate AA metabolism. The 12/15-LOX pathway was well studied in two types of mouse models of dermal fibrosis (tight skin model and bleomycin-induced mouse model) (Kronke et al., 2012), which revealed that 12/15-LOX-deficient mice have a higher susceptibility to dermal fibrosis than WT mice. Moreover, 12/15-LOX-deficient fibroblast cells are more responsive to TGF-β1 stimulation. These results fully proved that 12/15-LOX is a negative mediator of fibrosis.

Nitrated fatty acids (NFAs) could reverse myofibroblasts and enhance collagen uptake by alveolar macrophages in a mouse model of pulmonary fibrosis (Reddy et al., 2014). 8-Isoprostane, an oxidized lipid produced by oxidative stress, has been shown to be correlated with parameters of vascular damage and pulmonary fibrosis in SSc patients (Tsou et al., 2015). Different levels of metabolites involved in FA oxidation processes were observed in both the blood and immune cells (plasma and DCs) of SSc patients compared to healthy controls. Alternation of these metabolites increased the production of pro-inflammatory cytokines IL6 which further promote fibrosis process (Ottria et al., 2020).

### Potential Treatments Based on Lipid Metabolism in SSc

#### Statins

Vascular endothelial cells are a vital component of the vascular wall. Dysfunction of endothelial cells represents an early marker of multiple vasculopathy-like atherosclerosis and SSc. Statins could protect endothelial cells from various risk factors and enhance their function (Obama et al., 2004). Statin therapy downregulates chemokines and their receptors on endothelial cells, thus exerting an anti-inflammatory effect against vascular damage (Steffens and Mach, 2004). Endothelial-protective effects are dose- and duration-dependent in SSc (Kotyla, 2018). Statin administration leads to fibroblast apoptosis *in vitro* in models of fibrotic disorders (Tan et al., 1999; Rombouts et al., 2003).

#### FA Supplements

The beneficial antifibrotic and endothelial-protective effects of FAs (DHA, PEA and linoleic acid (LA)) were primarily explored in other fibrotic diseases (Chen et al., 2011; Bianchini et al., 2012; Kang et al., 2015). Downregulated matrix metalloproteinase-2 (MMP2), blocked mesenchymal-to-mesenchymal transition (MMT) and a reversed myofibroblast phenotype were revealed in DHA-exposed human prostate fibrocytes, thus inhibiting tumorigenesis (Bianchini et al., 2012). Lipid metabolism is largely downregulated in human kidney fibrosis samples, and deficiency of FA oxidation in tubule epithelial cells plays a critical role in metabolic reprogramming (Kang et al., 2015). Correcting lipid metabolism disorders effectively protects mice from tubulointerstitial fibrosis. FO supplementation can prevent cardiac fibrosis by activating the cyclic GMP/protein kinase G (cGMP/PKG) signalling pathway (Chen et al., 2011). Given that FO administration is well tolerated and safe in clinical practice, novel therapy trials should be applied to SSc.

## Lipid Metabolism Disorders in IIM

IIMs are chronic autoimmune myopathies characterized by skeletal muscle weakness and fatigue. The major subgroups of IIM are polymyositis (PM), dermatomyositis (DM), inclusion body myositis (IBM) and immune-mediated necrotic myopathy (IMNM) (Mariampillai et al., 2018).

### Dyslipidaemia in IIM

Dyslipidaemia is a common disorder in untreated DM patients and indicates a high risk of atherosclerosis (Wang et al., 2013). A negative correlation between CRP and HDL-C was found in DM patients, suggesting that inflammation may contribute to changes in the serum lipid profile. Sixty-five percent of juvenile DM patients were found to have quantitative subcutaneous fat loss, and 66% of these patients had hypertriglyceridemia (Verma et al., 2006). Whether both contribute to juvenile DM remains elusive. Cholesterol, low-density lipoprotein receptor (LDLR), very low-density lipoprotein receptor (VLDLR) and lipoprotein receptor-related protein (LRP) were increased in the muscle tissues of IBM patients (Jaworska-Wilczynska et al., 2002), which might be involved in the formation of vacuolated muscle fibres (VMF) by interacting with amyloid-β precursor protein (AβPP). The accumulation of LDLR and VLDLR may participate in the pathogenesis of IBM or repair and necrotizing processes.

### FAs in IIM

Accumulated evidence has revealed that AA metabolites (mainly leukotriene and prostaglandin subfamilies) are involved in skeletal muscle repair, proliferation and differentiation (Prisk and Huard, 2003; Sun et al., 2009). LTB4, secreted by neutrophils, macrophages, dendritic cells and mast cells, is a powerful chemokine that induces myeloid leukocytes and is a potential marker of activated T cell migration to inflamed muscle tissues (Page et al., 2004). The LTB4 pathway was found to be upregulated in the skeletal muscle tissues of PM/DM patients and negatively correlated with muscle weakness and fatigue (Loell et al., 2013).

Lymphocyte inflammation plays a critical role in the pathogenesis of PM (Dalakas, 2015), and mechanistic target of rapamycin (mTOR) signalling participates in this process. mTOR interacts with inflammation and metabolism and strongly controls *de novo* synthesis of palmitoleic acid (PA). Thus, upregulated PA was proposed to be a novel marker of PM (Yin et al., 2017).

### Potential Treatments Based on Lipid Metabolism in IIM

#### Anti-Rheumatic Drugs

Treatment with immunosuppressive agents in adult DM/PM patients can significantly dysregulate the expression of lipid metabolism-related genes (Loell et al., 2016), such as upregulation of FA uptake and transport genes (fatty acid-binding protein 7 and subfamily D member 2) and lipolysis genes (lipoprotein lipase, carboxylesterase 1 and hormone-sensitive lipase) and downregulation of anti-lipolysis genes (lipid storage droplet protein), which suggests enhanced generation of free FAs and intramuscular lipid accumulation, leading to skeletal muscle dysfunction.

### FA Supplements

Oral FO can improve muscle function and strength in elderly women and improve muscle weakness and fatigue in myositis patients (Rodacki et al., 2012). 5-Lipoxygenase activating protein (FLAP) is the determining leukotrienes synthesis protein (Back et al., 2007), including LTB4 synthesis (Borgeat and Naccache, 1990). However, immunosuppressive treatment of DM/PM cannot sufficiently suppress the LTB4 pathway, and FLAP inhibitors might be an ideal choice; this possibility requires further investigation.

## Lipid Metabolism Disorders in pSS

Primary Sjögren’s syndrome (pSS) is an autoimmune disease characterized by progressive lymphocytic infiltration into exocrine glands. pSS patients have a higher prevalence of metabolic disorders, such as dyslipidaemia and diabetes (Ramos-Casals et al., 2007; Kang and Lin, 2010). The relationship between metabolic disorders and pSS was first discovered in 1977 and described as ‘pseudo-SS’ (Goldman and Julian, 1977).

### Dyslipidaemia in pSS

Dyslipidaemia was positively correlated with minor salivary gland morphological changes in xerostomic patients (Lukach et al., 2014). These altered lipid profiles are associated with high levels of ESR (Cruz et al., 2010), and hypercholesterolemia is negatively correlated with immunological markers (complement C3, complement C4, anti-Ro and anti-La) (Ramos-Casals et al., 2007). Thus, lipid profiles might be valuable for evaluating disease activity.

### FAs in pSS

A link between palmitic acid levels in the blood and the pathogenesis of pSS was revealed (Shikama et al., 2013). Palmitic acid is involved in the differentiation of CD4^+^ T cells and induces IL-6 production (Rincon et al., 1997), thus promoting local inflammation and monocyte infiltration in the salivary glands (Sekiguchi et al., 2008). High levels of palmitic acid can also induce epithelial cell apoptosis in the salivary glands (Miller et al., 2005), leading to an elevated level of α-fodrin fragment, which is an auto-antigen in the pathogenesis of pSS (Haneji et al., 1997). In addition, a high-fat diet leads to advanced inflammation in the salivary glands and an elevated titre of auto-antibodies in a mouse model of pSS (Haneji et al., 1994).

### Potential Treatments Based on Lipid Metabolism in pSS

#### Anti-Rheumatic Drugs

In pSS patients, hydroxychloroquine (HCQ) administration reverses the disorder of TG and HDL levels (Migkos et al., 2014), which are strongly correlated with the risk of atherogenesis. This implies that dyslipidaemia is a specific symptom in the pSS population, rather an independent risk factor for atherogenesis.

#### FA Supplements

Lipid-related molecules are beneficial for the salivary glands both *in vitro* and *in vivo*. DHA can inhibit palmitic acid-induced IL-6 and IL-8 production (Shikama et al., 2015). The RvD1 biosynthetic pathway was shown to exist in murine and human salivary gland cells (Leigh et al., 2014), and its biosynthesis-related mediators are quite different in salivary gland cells from pSS patients than in those from normal controls. RvD1 can inhibit TNF-α-mediated inflammation, increase cell polarity and enhance the barrier function of salivary glands (Odusanwo et al., 2012; Nelson et al., 2014). Therefore, DHA supplementation may be a novel therapy for pSS patients (Table 1).

**TABLE 1:** Dyslipidaemia in rheumatic diseases.

Rheumatic diseases	Dyslipidaemia	Functions	Reference
SLE	piHDL ↑	Positively correlated with carotid plaque and IMTs	McMahon et al. (2006), Wu et al. (2016)
	HDL, Apo-A1↓, ox-LDL↑	Observed in paediatric SLE patients	Soep et al. (2004), Navab et al. (2004)
	PON-1↓	Protect LDLs from oxidation	Kiss et al. (2007)
	LDL ↑	Positively correlated with plaque inflammation	Navab et al. (2004)
	Apo-B,TG,TC↑	Observed in LN patients	Hahn (2010), Liu et al. (2014)
RA	Pro-oxidant HDL ↑	Positively correlated with ESR, hsCRP and DAS28	McMahon et al. (2006), Gomez Rosso et al. (2014)
	Ox-LDLs ↑	Positively associated with RA disease activity, IMTs	Charles-Schoeman et al. (2012), Kim et al. (2004)
SSc	HDL-C↓, LDL↑	Negative correlated with carotid artery IMTs	Denton and Khanna (2017)
	Lipoprotein(a) ↑	Positively correlated with vascular damage	Tsifetaki et al. (2010)
	Ox-LDL/β2GPI complex ↑	Positively correlated with autoimmune vascular inflammation	Lippi et al. (2006)
IIM	HDL-C↓	Negative correlated with CRP and inflammation	Mariampillai et al. (2018)
	Cholesterol, LDLR, VLDLR, LRP↑	Involved in the formation of vacuolated muscle fibres	Wang et al. (2013)
pSS	Cholesterol↑	Negatively correlated with immunological markers	Back et al. (2007)

## Conclusion

Altered lipid profiles are common in rheumatic diseases. Dyslipidaemia, a traditional risk factor for atherosclerosis, participates in the development of rheumatic diseases. The coexistence of rheumatic diseases and atherosclerotic diseases increases the mortality of rheumatic diseases. Statin agents not only lower atherosclerotic risk but also seem to be immunomodulators of rheumatic diseases. FA metabolism also plays critical roles. How altered FAs and their metabolites regulate inflammation and exert other specific effects remains unknown. Vascular damage in autoimmune diseases is partly caused by the oxidation of FAs and their metabolites. Lipid metabolism can directly influence T cell (de Jong et al., 2014) and macrophage (Galvan-Pena and O'Neill, 2014) function. CTLA-4, ICOS molecules and lipid synthesis pathways are defective in raptor-deficient regulatory T (Treg) cells (Zeng et al., 2013).

Little is known about the interactions among lipid metabolism, immune cell function and rheumatic diseases. Immunometabolism may be vital in the development of rheumatic diseases. Given the importance of lipid profiles and metabolism, further investigations about mechanism and therapeutic strategies are urgently needed.

## Glossary

ATF-3: activating transcription factor 3
AβPP: amyloid-β precursor protein
BAL: bronchoalveolar lavage
cGMP/PKG: cyclic GMP/protein kinase G
CRP: C-reactive protein
CVD: cardiovascular disease
DAS28: disease Activity Score in 28 joints
DM: dermatomyositis
dWAT: dermal white adipose tissue
ESR: erythrocyte sedimentation rate
FA: fatty acid
FABP4: fatty acid-binding protein 4
FAT: fatty acid translocase
FFA: free fatty acids
FO: fish oil
HCQ: hydroxychloroquine
HDL: high-density lipoprotein;
hsCRP: high sensitivity C-reactive protein
IBM: inclusion body myositis
IFN-γ: interferon-γ
IIM: idiopathic inflammatory myopathy
IMNM: immune-mediated necrotic myopathy
IMT: intima-media thickness
LDL: low-density lipoprotein
LDLR: low-density lipoprotein receptor
LN: lupus nephritis
LPL: lipoprotein lipase
LRP: lipoprotein receptor-related protein
LTB4: leukotriene B4
LTE4: leukotriene E4
LXRα: liver X receptor α
MHC: Class II major histocompatibility complex
MMP2: matrix metalloproteinase-2
MMT: mesenchymal-to-mesenchymal transition
mTOR: mechanistic target of rapamycin
MTX: methotrexate
oX-LDL: oxidized LDL
PBMCs: peripheral blood mononuclear cells
PGD2: Prostaglandin D2
PGE2: Prostaglandin E2
PGI2: Prostaglandin I2
pi-HDL: Pro-inflammatory HDL
PM: polymyositis
PON-1: Paraoxonase-I
PPARγ2: peroxisome proliferator activated receptor-γ2
PPP: the pentose phosphate pathway
pSS: Sjögren's syndrome
PUFAs: polyunsaturated fatty acids
RA: rheumatoid arthritis
RANKL: receptor activator of nuclear kappa B ligand
RASF: RA synovial fibroblasts
RvD1: Resolvin D1
SLE: systemic lupus erythematous
SLEDAI: SLE disease activity index
SSc: systemic sclerosis
TC: total cholesterol
TCA: the tricarboxylic acid
TG: triglyceride
TLR: Toll-like receptor
VLDLR: very low-density lipoprotein receptor
VMF: vacuolated muscle
